# Love songs and serenades: a theoretical review of music and romantic relationships

**DOI:** 10.3389/fpsyg.2024.1302548

**Published:** 2024-02-14

**Authors:** Joshua S. Bamford, Julia Vigl, Matias Hämäläinen, Suvi Helinä Saarikallio

**Affiliations:** ^1^Centre of Excellence in Music, Mind, Body and Brain, University of Jyväskylä, Jyväskylä, Finland; ^2^Institute of Human Sciences, University of Oxford, Oxford, United Kingdom; ^3^Department of Psychology, University of Innsbruck, Innsbruck, Austria

**Keywords:** mate choice, romantic (love), dance, social bonding hypothesis, human evolution

## Abstract

In this theoretical review, we examine how the roles of music in mate choice and social bonding are expressed in romantic relationships. Darwin’s Descent of Man originally proposed the idea that musicality might have evolved as a sexually selected trait. This proposition, coupled with the portrayal of popular musicians as sex symbols and the prevalence of love-themed lyrics in music, suggests a possible link between music and attraction. However, recent scientific exploration of the evolutionary functions of music has predominantly focused on theories of social bonding and group signaling, with limited research addressing the sexual selection hypothesis. We identify two distinct types of music-making for these different functions: music for attraction, which would be virtuosic in nature to display physical and cognitive fitness to potential mates; and music for connection, which would facilitate synchrony between partners and likely engage the same reward mechanisms seen in the general synchrony-bonding effect, enhancing perceived interpersonal intimacy as a facet of love. Linking these two musical functions to social psychological theories of relationship development and the components of love, we present a model that outlines the potential roles of music in romantic relationships, from initial attraction to ongoing relationship maintenance. In addition to synthesizing the existing literature, our model serves as a roadmap for empirical research aimed at rigorously investigating the possible functions of music for romantic relationships.

## 1 Introduction

Love and romance are pertinent and ubiquitous topics in music across cultures and centuries (Mehr et al., 2018). Meanwhile, music itself appears to be universally present in all cultures in some form (Brown, 1991; Brown and Jordania, 2013; Savage et al., 2015; Mehr et al., 2019), This ubiquity, along with the fact that music seems to be largely absent in our primate relatives (Merker, 1999; although see Raimondi et al., 2023), suggests that music may have evolved in early humans. Yet, romantic love has not been at the centre of the academic investigation on why the human capacity for music might be an adaptive feature for our species.

The recent scientific literature on the evolutionary function of musicality has primarily focused on theories of social bonding (e.g., Roederer, 1984; McNeill, 1997; Freeman, 1999; Peretz, 2006; Dunbar, 2012; Launay et al., 2016; Harvey, 2017; Patel, 2018; see Savage et al., 2021a for review) and coalition signaling (e.g., Hagen and Bryant, 2003; Mehr et al., 2021). Both of these accounts are broadly compatible with the notion that musicality was primarily used in parent-infant interactions, and only later to support adult musicking (e.g., Cross, 2001; Leongómez et al., 2022). An older strand of theoretical accounts has explored sexual selection as an explanation for human musicality (e.g., Miller, 2000), following the ideas first articulated by Darwin (1871) in The Descent of Man. This line of thinking notes that, for example, popular musicians are often portrayed as sex icons, seemingly supporting the notion that musicality may be attractive and could be a factor affecting mate choice (Marin and Rathgeber, 2022).^1^

Overall, the sexual selection and social bonding approaches suggest two different ultimate functions of music: music for attraction and music for connection. Music for attraction may be used to display either general fitness or compatibility with a potential partner (Rose et al., 2022), while music for connection serves the social bonding functions described by Savage et al. (2021a), as part of a tradition of social bonding mechanisms exhibited in other primates, including duetting behavior in some other species (Raimondi et al., 2023). Music may therefore be used to attract potential partners, but also to reinforce and maintain bonds with those partners.

At first glance, these two theories of the evolutionary functions of music - social bonding and sexual selection - may seem to be disconnected. However, a closer look reveals that these apparently distinct functions may be intimately related within the context of romantic relationships. In seeking to bridge the gap between the evolutionary functions of music and the nuanced dynamics of romantic love, we recognize the need for a more holistic approach. Beyond mere sexual attraction and passion, romantic love encompasses a rich spectrum from self-expansion to companionship. This complexity positions romantic love as an ideal context for addressing the theoretical accounts of music’s evolutionary function dialogically.

We therefore propose a theoretical framework that explains how music can be used across the stages of romantic relationships - attraction, formation, and maintenance - to enhance the three fundamental components of love according to Sternberg (1986): passion, intimacy and commitment. In doing so, we will look at both the proximate and ultimate mechanisms behind music and love, and how they may overlap and relate to each other. To be clear, we are not suggesting that the only function of music is to be found in romantic relationships, but rather that there may be multiple evolutionary functions of music, and that some of these functions may still be seen in romantic relationships.

While the sexual selection theory has been partially tested in the context of dating (e.g., Marin and Rathgeber, 2022), social bonding theory has been comparatively little tested in romantic contexts (although see Sharon-David et al., 2019), but mainly with strangers, friends or groups (e.g., Weinstein et al., 2016). This raises the question of whether the mechanisms behind music’s bonding effects are the same for all types of relationships, or whether there are specific effects for the development and maintenance of romantic love. The social bonding effects of music may be most closely associated with intimacy, but romantic love also involves passion and commitment (Sternberg, 1986). It is therefore worth exploring the specifics of how the sexual selection and social bonding hypothesis for music might relate to existing theories of love and relationships in humans. We can then ask: are the effects of music in romantic relationships different from the general social bonding effects of music? How might these two proposed functions of music (for attraction and bonding) be related? And finally, are these dual functions expressed differently in different stages and types of relationships? Answering these questions may provide new insights into how the evolution of music and musicality may have been shaped by love.

In what follows, we will explore the biological parallels between music and romantic love, delving into the proximate and ultimate mechanisms of both. We then present a novel theoretical model that attempts to unify these seemingly divergent theories and highlights music’s potential to enhance different components of romantic love at different stages of a romantic relationship. Our aim here is to develop a theoretical account that may be tested with future empirical study, rather than to settle any debate over the ultimate functions of music(ality).

Before moving on to the main parts of this article, a note on the concept of music is in order. In this text, we use the term music in a very broad sense, including literature on musicality, music preferences, listening to music, making music together, and dancing. The foundational abilities required to make music (referred to collectively as musicality) must have necessarily preceded music itself (Honing et al., 2015). We note that musicality is not a monolithic ability, but is instead made up of many component abilities (Fitch, 2015). These different components may serve different functions; e.g. rhythm may be primarily for social synchrony, while pitch is for emotion expression (see Leongómez et al., 2022). Some of these abilities may have evolved specifically to enable musicking, because of the benefits that musicking brings, but many may be the product of other selection pressures and later incorporated into musicality (i.e., the ‘cheesecake hypothesis’; Pinker, 2003). Regardless of how any specific component of musicality emerged, once humans started making music, this may have created a new cultural environment that selected for specific musical abilities, in a process of gene-culture coevolution (Patel, 2018). The extent to which these abilities are unique to supporting musical behavior is a matter for later discussion. As the human social environment is constantly changing, it is possible that some selection pressures for musicality have changed, and that music no longer serves a function that it once did; musicality is not one thing serving one function across all cultures at all times in history. This paper is discussing the use of music in contemporary romantic relationships, therefore we are primarily concerned with the functions that musicality may still serve today in such relationships. Nevertheless, we also consider music-like behaviors in humans and other species to be relevant for this discussion, including birdsong, infant-directed song and infant-directed speech (parentese), as they relate to the phylogeny and ontogeny of modern, adult musicking.

Finally, another important question may arise: Why was music chosen as the focus of this review? Is music more effective than alternative activities in generating mutual attraction, assessing compatibility, and fostering closeness in different stages of relationships? It is important to clarify that our investigation of the specific role of music in romantic relationships does not exclude the potential for other leisure activities to produce comparable effects. Even if music may be an evolutionarily ancient social bonding mechanism (Savage et al., 2021a), a wide range of modern activities may have replaced some of those functions, or be more effective specifically for romantic relationships. However, we suggest that music still has specific characteristics that make it uniquely suited to facilitate various dimensions of love, particularly through elements such as coordination, synchronicity, and divided attention (e.g., Harwood and Wallace, 2021). In what follows, we provide examples that illustrate how music may contribute to relationship formation and maintenance, a proposition that requires empirical testing in future studies.

## 2 Evolutionary biology of music and love

The social bonding effects of music have gained increasing attention in the scientific literature. Engaging in musical activities and other tasks that require interpersonal synchronization has consistently been found to increase feelings of social closeness and promote prosocial behavior (e.g., Hove and Risen, 2009). This had led to a suggestion that the social bonding effects of music may have contributed to the evolution of human musicality (see Savage et al., 2021a, for a review): music is then used as a tool to encourage interpersonal synchronization, which promotes social bonding, and groups and/or individuals who are better able to form bonds within a group may have a selective advantage, therefore leading to a selection pressure for sensorimotor synchronization which is a fundamental skill for musical behavior. This so-called synchrony-bonding effect invokes (multilevel) natural selection, and may explain why adult humans make music with each other. However, it is often presented with the exclusion of sexual selection, and has rarely been discussed in terms of romantic relationships. This is despite frequent observations of love songs across a wide range of cultures (Mehr et al., 2018, 2019) and much anecdotal evidence of music and dance being used to find romantic partners, not just to bond with friends. There is potentially scope to extend the theoretical understanding of the evolutionary function of music to include such relationships.

The evolution of romantic love appears to share much in common with the evolution of adult musicking. Both have an evolutionary history that may have involved co-opting mother-infant bonding mechanisms (e.g., Leongómez et al., 2022; Nguyen et al., 2023), and both appear to engage similar neurohormonal systems (e.g., Tarr et al., 2014; Harvey, 2020). Music and love both appear to be universal across human cultures (e.g., Brown and Jordania, 2013; Sorokowski et al., 2021), which has led numerous theorists to suggest adaptive functions. As discussed in what follows, the possible adaptive functions of music can be discussed in terms of both sexual selection (through attraction and mate choice) and multilevel natural selection (through group bonding and coalition signaling). Meanwhile, the emotions and behaviors associated with love - such as feelings of attraction, attachment, commitment, and caregiving - may have evolved to ensure the successful reproduction and survival of offspring (Campbell and Ellis, 2005; Fletcher et al., 2015; Buss, 2018).

It is important for any evolutionary explanation to consider Tinbergen’s (1963) four questions regarding the causation, ontogeny, phylogeny and function of a behavior (see Fitch, 2015). For instance, the evolutionary history of music as parent-infant communication addresses the question of ontogeny, while measuring hormone responses to music provide mechanistic explanations. There have been previous attempts to describe romantic love in Tinbergian terms (Bode and Kushnick, 2021), which we will briefly recapitulate here in comparison with music.

### 2.1 Causation—what are the mechanisms underlying music and love?

*‘At its most basic level, love is biological bribery. It is a set of neurochemicals which motivate you to, and reward you for, commencing relationships with those in your life who you need to cooperate with—friends, family, lovers, the wider community—and then work to maintain them.’* (Machin, 2022, p. 21).

Numerous social, emotional and psychological mechanisms are involved in the experience of love. These are underpinned by neurohormonal pathways that may have evolved to motivate loving behavior, bond partners to each other, and reduce the search for other partners (Bode and Kushnick, 2021). Different social dispositions, dyadic relationships and wider social network relationships may be associated with different hormones, including: endorphins, oxytocin, vasopressin, dopamine, serotonin and testosterone (Pearce et al., 2017). Of these, endorphins (particularly the β-endorphin system) and dopamine appear to be relevant to all social domains, while oxytocin is primarily involved in reproductive relationships (i.e., romantic bonding and parent-infant bonding; Pearce et al., 2017). However, the oxytocin, endorphin, and dopamine systems appear to influence each other (George and van Loon, 1982; Putnam and Chang, 2022). A lot of work has been done on pair bonding in prairie voles, a monogamous species, finding that reward systems in the brain create positive associations with the partner, built upon the oxytocin and dopamine systems (Walum and Young, 2018). Meanwhile, work on primates highlights the role of endorphins in both romantic and friendship bonds (Machin and Dunbar, 2011). Both endorphins and dopamine are part of the brain’s reward system, suggesting that we have systems that reward us for being social (Pearce et al., 2017). Crucially, the release of endorphins can be triggered by other individuals through social activity.

Although there is disagreement about the exact mechanisms by which the synchrony-bonding effect works, it appears to involve the same hormonal systems that are related to social bonding. Making and listening to music have been shown to activate the oxytocin system, which is also involved in parent-infant bonding in most mammals, and appears to have a wide range of social effects in humans (Harvey, 2020). In addition, experimentally manipulating oxytocin levels increases performance on synchronized tasks (Gebauer et al., 2016; Josef et al., 2019). Synchrony also engages the same endorphin reward system that is also active during social grooming in primates (Cohen et al., 2010; Tarr et al., 2014). It is not yet understood precisely how synchrony hijacked this reward system, although it could be because of a processing fluency response to synchronized stimuli (Bamford, 2022; Bamford et al., 2023), and processing fluency is generally rewarding (Winkielman et al., 2003; Landwehr and Eckmann, 2020).

There are many activities that engage both the oxytocin and endorphin reward system. These include social touch, shared laughter, and synchronized action—including music and dance (Cohen et al., 2010; Dunbar, 2010, 2012, 2022; Nummenmaa et al., 2016; Handlin et al., 2023). As discussed above, the rewarding experience of engaging in musical activity may underpin the social bonding effects of music, and this appears to engage similar mechanisms to those behind love and social bonding more broadly.

### 2.2 Ontogeny—how do music and love develop during lifetime?

The second question concerns the development of love and musical ability across the lifespan. Passionate love begins as early as three or four years of age (Hatfield et al., 1988), but reaches full maturity in adolescence (Hatfield and Sprecher, 1986). Any specific experience of romantic love tends to progress through the relationship stages discussed above, and usually ends either in separation or in progression to companionate love. Many factors seem to influence preferences, including: personality, appearance, proximity, perceived similarity, mutual attraction, similarity to one’s parents (Pines, 2005). There may be cultural differences in the relative importance of each of these factors (Bode and Kushnick, 2021), suggesting some role for developmental circumstances. In particular, prior experiences of parent–child attachment are thought to shape later expectations of romantic relationships, as expressed in adult attachment style (Hazan and Shaver, 1987).

Similarly to love, we begin developing musical abilities from a very early age. It has been argued that the earliest musical experiences occur *in-utero*, as a fetus can hear its mother’s voice and other environmental sounds, which it can then learn to associate with its mother’s emotional state (Parncutt and Chuckrow, 2019). This has been extended to suggest that rhythm processing in particular, may develop from experiencing one’s mother walking *in-utero* (Larsson et al., 2019). From birth, infant-directed song and dance become important tools for directing infant attention and for parent-infant bonding (Feldman, 2007; Nguyen et al., 2023), as is infant-directed dance (Kim and Schachner, 2023). The boundaries between music and language are blurred in these early-life interactions, as infant-directed speech is more melodic, rhythmic and ritualized than ordinary speech, making it much more music-like (Dissanayake, 2004; Saint-Georges et al., 2013). These early life experiences of interpersonal synchrony with a parent may then establish skills that are used for music and dance in adulthood (Phillips-Silver and Keller, 2012). Indeed, children appear to learn much about empathy through music (Rabinowitch et al., 2013). This is similar to how early experiences of love shape preferences and behavior later in life.

### 2.3 Phylogeny—how did music and love evolve?

The evolutionary history of love may be based on independent emotional systems and the co-optation of mother-infant bonding mechanisms (Bode and Kushnick, 2021). Evidence for this can be found in pair bonding behavior in non-human primates, as well as in observations of modern humans. Fisher (2000) suggests that love involves three independent emotion systems: sex drive (lust), attraction (romantic love), and attachment (pair-bonding). Each of the three may be associated with different neurohormonal mechanisms, and different adaptive functions. Fisher’s three emotion systems somewhat overlap with Sternberg’s (1986) triangle model of love (as discussed in section 3 and in the Supplementary material), although such parallels are imperfect and both authors use slightly different definitions. For instance, sex drive and attraction in Fisher’s model may both relate to different factors of passion in Sternberg’s model, while commitment—being a ‘cold’ component of love—may not relate as closely to Fisher’s emotional systems. Fisher (2006) notes that ‘attraction’ in humans shares many parallels with attraction and courtship in other mammals, suggesting that this may be an ancestral mechanism.

Although some of the emotion systems used in romantic love may be very old, others may have developed their modern function relatively recently. The origins of pair-bonding and attachment may lie in mother-infant bonding. It has been noted that neural activation is very similar in both romantic and maternal love (Bartels and Zeki, 2004). Mother-infant bonding is present in many more species, and these pathways may have been co-opted to promote pair-bonding in monogamous mammals (Numan and Young, 2016).

Humans and some other primates may have taken this a step further, as the mechanisms that have been co-opted for pair-bonding are also used to maintain relationships with friends and extended family, rather than just mates and offspring (Shultz and Dunbar, 2007; Dunbar, 2018). Most non-human animals do not invest as much time and energy in maintaining friendships—although see Emery et al. (2007) regarding pair-bonding in birds. This evolution of attachment mechanisms in humans may be closely related to the evolution of alloparenting and communal child-rearing through familial ties (Fletcher et al., 2015).

Just as romantic love may have co-opted adaptations for mother-infant bonding (Bode and Kushnick, 2021), the bonding effects of music may also have originated in infant-directed song (Nguyen et al., 2023). Infant-directed song appears to be ubiquitous across cultures, particularly in the form of lullabies (Mehr et al., 2018), and spontaneous music-induced movement has been observed as early as 5 months old in human infants (Zentner and Eerola, 2010). Although ontogeny rarely recapitulates phylogeny (Keil and Newman, 2010), in the case of music and love, both seem to have their developmental and evolutionary origins in parent-infant bonding.

Both romantic love and musicality may also have co-opted more general emotion systems. This is evident in Fisher’s (2000) model of emotion systems in romantic love outlined above. However this may also be the case in music. Musicality consists of many component abilities that are unlikely to have evolved for a unified purpose (Fitch, 2015). Many of the emotional effects of music seem to engage domain-general processes for emotion regulation, particularly seen in the use of lullabies to calm infants or dance music to excite and coordinate groups (Singh and Mehr, 2023). These emotion systems likely did not evolve specifically for music or love, but are exploited by them. Given the parallels between music and romantic love—the overlapping mechanisms and similar functions—it is worth exploring how else they might be related. In particular, how music might be used in romantic relationships at each of the stages outlined in the previous section.

### 2.4 Functions—what are the survival advantages of music and love?

Having established the mechanisms behind love and music, and how they develop across the lifespan, the next question is to ask why humans make music and fall in love in the first place. It must be acknowledged that functional explanations can easily turn into ‘just so’ stories that can be almost impossible to empirically test. Nor is it true that all traits necessarily need to have a function, as they may be a byproduct or the result of random genetic drift. In the case of music and love, this seems unlikely. Both take significant time and effort that could otherwise be devoted towards an individuals own survival, meaning that there should be a strong selection pressure against them if they provided no other benefits; traits with no function are usually only preserved if they are benign. The benefits of love may be more immediately obvious, but there is also substantial evidence for some function of music, as discussed below.

#### 2.4.1 Musicality and mate choice

The first suggestion of a role for sexual selection in the evolution of musicality was made by Darwin, 1871 in The Descent of Man, where he compared music to birdsong as a sexual display. He proposed two forms of sexual selection (Darwin, 1871; Rosenthal and Ryan, 2022): intersexual selection (e.g., birds calling to attract a mate) and intrasexual selection (e.g., male competition for mates, such as in deer). Most theories of music have focused on the former (mate attraction and mate choice). Our model presented in section 3.2 continues this trend, as it deals with romantic couples and not potential rivals outside the partnership.^2^ It should also be noted that Darwin’s theories may have been influenced by personal experience and the historical context in which he lived. Victorian norms around romantic relationships certainly influenced his theories about mate choice in humans, leading him to downplay the role of females (Ryan and Jetha, 2010, p. 28; Rosenthal and Ryan, 2022). Music was also an important part of Darwin’s family life, and he was reportedly very attracted to his wife’s musical ability (Bannan, 2014, 2020), which may have further shaped his views on the sexual function of musicality. Nevertheless, research on sexual selection in humans has progressed since Darwin’s work in this area.

Signaling attractiveness between potential partners is an important process in mate choice. Darwin’s original theory supposed that the exact traits being selected could be completely arbitrary. Under this model, the only selection pressure is the esthetic preferences of the sex (usually female) doing the selecting (Prum, 2012). This was later rationalized by Zahavi’s (1975) handicap principle, stating that any individual that could sustain traits that only had costs but no benefits must have excess fitness. However, cues can also be considered honest signals if they are too difficult to fake (Biernaskie et al., 2014), rather than just being costly. A trait that is associated with increased survival (of oneself or one’s offspring) can be an honest signal of evolutionary fitness that is experienced as attractive by the perceiver (Lewis et al., 2022). Sexual selection of this kind is then really an extension of natural selection in which potential mates are selecting traits that may indicate some adaptive benefit (Wallace, 1895; Prum, 2012). There are various candidates for traits selected by potential mates in humans, but what about musicality?

In contrast to Darwin’s original theory of sexual selection, most authors writing about the sexual selection of music have primarily considered music as an honest signal of general fitness, and have focused on intersexual mate choice. For instance, Miller (2008) suggested that many human behaviors, including music, are honest markers of general intelligence, and general fitness (Miller, 2008). Creativity may indicate a well-functioning nervous system, which would have other survival benefits (Watkins, 2017; Novaes and Natividade, 2023). If general creativity is a marker of fitness, then musical creativity may be likewise (Miller, 2000).

However, the expectation that musicality would be universally considered attractive or associated with mating success has yielded conflicting results. For example, one large study (Mosing et al., 2015) did not support an association between music aptitude, musical achievement and mating success. Meanwhile, some studies with smaller samples (e.g., Tifferet et al., 2012; Madison et al., 2018; Marin and Rathgeber, 2022) have presented cases in which displays of musicality increased mate value and attractiveness, although these did not measure mating success directly. One review found limited evidence that musicality increases mating success, although neither mating success nor attractiveness are necessarily tied to reproductive success (i.e., survival of offspring; Ravignani et al., 2017). It could be that parents’ musicality may improve developmental outcomes for their offspring (Leongómez et al., 2022). However, this would not explain why adults make music together, unless musicality could be a cue for parental ability, or a means of practicing with a partner in preparation for raising offspring.

Typically, sexually selected traits are also sexually dimorphic, such as the peacock’s tail (Padian and Horner, 2014), which would be expected also for music in the case of its relevance for sexual selection. A clear example of sexual dimorphism is in the human singing voice. Males and females vocalize around an octave apart, which may be the result of both historical intrasexual competition that has led male primates to exaggerate their size through deeper voices, and the need for early human males to harmonize with females and children (Bannan et al., 2022). Studies have shown that choir boys even modulate their timbre to make individual voices stand out more when females are present (Keller et al., 2017), suggesting a tension between blending in and standing out. This duality of function may be an important factor in the evolution of human musicality, and a reminder that different components of musicality may have evolved for different functions at different times.

There is some evidence to suggest that there are sex differences in musical preference, specifically that female musical preferences change with the menstrual cycle (Ravignani et al., 2017). This supports the sexual selection hypothesis, as perception of sexually selected features tend to peak around ovulation (Charlton, 2014). Female auditory perception indeed seems to vary across the menstrual cycle, with better performance on auditory discrimination tasks, temporal perception, speech-in-noise perception and auditory working memory (e.g., Sao and Jain, 2016; Carneiro et al., 2019). Other studies have examined women’s preference for complex music around ovulation, some with evidence (e.g., Charlton, 2014), some without (e.g., Charlton et al., 2012).

Meanwhile, Marin and Rathgeber (2022) found that, while presenting opposite-sex individuals as performers of music increased dating desirability for both males and females, only females’ ratings of male facial attractiveness were enhanced by music. Some authors also suggest that females have better melody recognition than males in general (Miles et al., 2016). Female listeners also exhibit greater pupil dilation, an indicator of increased psychological arousal, when listening to groovy music (Bowling et al., 2019). Misattribution of this arousal from the music to a person may be one potential mechanism through which music enhances attractiveness (Marin et al., 2017).

In another study, single female Facebook users responded more positively to friend requests from a male user with a guitar in his profile picture than from the same male user without a guitar in his profile picture (Tifferet et al., 2012); although this is really a test of attractiveness through association with a cultural symbol, and not from musical ability.

Another useful cue to attractiveness are dance movements (Fink et al., 2015), and there may also be sex differences in how people judge the attractiveness of others based on their dance moves (Luck et al., 2012). The use of dance in courtship rituals is common in many cultures, often with males displaying to females, such as in the *yaake* dance for the Wodaabe people or in Swedish *polska* (Kaminsky, 2011; Curnow, 2018). This also highlights how any study of music and attractiveness should consider the cultural context, as there may be learnt preferences that should be taken into account.

However, the evidence for sexual dimorphism in music making or music preferences in humans is not as clear-cut as in species where only one sex performs and the other perceives. Some studies have found conflicting or negligible evidence for differences in general musical aptitude in males and females (e.g., Zentner and Strauss, 2017; Bertolo et al., 2023). Furthermore, music itself does not seem to be perceived as inherently gendered (Sergeant and Himonides, 2014). Meanwhile, some high-profile studies that did make bold claims about musicality and sexual attraction were later retracted (e.g., Brown et al., 2005; Guéguen et al., 2014). Taking the evidence into account, some authors have argued that there is little sexual dimorphism for musicality (Mehr et al., 2021), although there clearly are some notable differences (e.g., in perceptual preferences and vocalizing pitch), and lack of dimorphism in other domains does not necessarily mean that there has been no role of sexual selection.

Contemporary sexual selection theory questions the extent to which stable sexual dimorphism should be expected in humans, finding instead a great deal of cultural variation in mate preferences and mate choice between the sexes (Kokko and Kempenaers, 2002; Brown et al., 2009). Firstly, genes for general intelligence and creativity are complex and not restricted to the Y chromosome, so sexual dimorphism would not necessarily be expected (Miller, 2008). Furthermore, sexual selection through mutual mate choice may be more common in humans, because both parents invest more in the offspring, which would not lead to the runaway sexual selection seen in some other species (Stewart-Williams and Thomas, 2013). Even in birds, the oft-cited sexual dimorphism in singing may be an adaptation to European and North American climates, while ancestral songbirds likely possessed more equal singing abilities between the sexes, albeit for different functions (Odom et al., 2014). Even in a species of duck, for which female mate choice seems more important, both partners need to be able to complete a head-bobbing courtship ritual (a kind of head-banging partner dance), and thus both sexes possess the required coordination abilities (Silberstein, 1983). In species with mutual mate choice, such as humans, both sexes need to be able to perceive and evaluate esthetic displays, so less sexual dimorphism would be expected (Miller, 2001; Varella, 2023). As Leongómez et al. (2022) point out, sex differences could be highly contextual, and may only exist in specific mating behavior, not in general ability. Thus, while research on sexual dimorphism in some domains of musicality remains inconclusive, this is not necessarily a death knell for the sexual selection hypothesis.

It is important also to consider that mating displays may also be about similarity and compatibility, not just general fitness. Both similarity and general fitness can be perceived as attractive to a potential partner (Rosenthal and Ryan, 2022). Animal studies have found that there is often a preference for familiarity over general fitness (Pfaus et al., 2012), and human preferences for scent can be based on scent markers of both general fitness and immune system compatibility (Wedekind et al., 1995; Thornhill, 2003). Therefore, any study of attractiveness and music should take into account the extent to which music signals general fitness or compatibility. Furthermore, given the relative lack of sexual dimorphism in musicality, it is possible that music plays a role in mutual mate choice based on both compatibility and general fitness, rather than one sex necessarily doing the selecting. It is also not uncommon for individuals to make sexual displays to an existing mate, to signal investment in the relationship (Servedio et al., 2019). Thus, early humans may have used their voices to signal dominance, but they may also have sung to signal general fitness through a display of creativity, and may continue to do so to maintain existing relationships.

#### 2.4.2 Social bonding through musicking

In recent years, more attention has been paid to the social bonding theory of the evolution of musicality (Savage et al., 2021a). This is based on research into social bonding in primates, which suggests that social touch and grooming are important in creating and maintaining social bonds within a group (Dunbar, 2010). There are many benefits to living in large, complex groups, and it has probably been a driving force behind increased cognitive abilities in humans (Shultz and Dunbar, 2007). However, increased sociality is also a source of stress that can impact upon fertility rates and needs to be mitigated (Dunbar, 2010; Dunbar and Shultz, 2021). Conversely, greater social cohesion increases survivability and reproductive fitness of the group (Richerson and Boyd, 2008; Wilson, 2012). Mechanisms to maintain that group cohesion, such as social grooming, are thus important for survival.

An inherent limitation of traditional social grooming lies in its time-intensive nature, as it can only be performed on a one-to-one basis, which is not scalable to larger groups. As human groups have grown in size, alternative behavioral mechanisms for bonding and stress alleviation emerged. This sequence of adaptations started with shared laughter, which is thought to have been a precursor to musical dancing (Dunbar, 2012, 2022). Some have argued that the primary survival benefit of musicality is in coalition signaling to those outside of the group (Mehr et al., 2021). However, this may be best understood as a component of the social bonding hypothesis: an honest signal is one that is difficult to fake (Biernaskie et al., 2014), so if synchronized behavior inevitably leads to social bonding, then this only strengthens the usefulness of synchrony as an index signal. Conversely, if it were easy to synchronize with someone without any increase in bondedness, then it may be too easy to fake a coalition.

It’s essential to recognize that musicality is not a singular, abrupt emergence but rather a composite of gradually evolving component abilities (Fitch, 2015). Within this realm, rhythm perception seems to be particularly relevant to the unifying effects of music (Leongómez et al., 2022). The synchronized actions involved in music-dance have been highlighted as a critical element in fostering social bonds (Rennung and Göritz, 2016; Vicaria and Dickens, 2016; Mogan et al., 2017), and these bonding effects may be shared with other synchronized behaviors (McNeill, 1997). Interestingly, this synchrony effect is geared towards strengthening social bonds rather than mere pro-social behavior (Tarr and Dunbar, 2023), and it seems to be present from a very young age (Tunçgenç and Cohen, 2018). Mechanical synchrony alone can produce bonding effects, but these effects appear to be enhanced by the presence of music (Stupacher et al., 2017). Synchronizing with music through dance has been shown to promote inter-brain synchrony (Basso et al., 2021), which indicates shared attention and cognition through completing a shared task. The presence of music provides a social ‘scaffolding’, helping to coordinate actions and bring people into synchrony (Tarr, 2017). Therefore, sensorimotor synchronization skills may have evolved as a means of maintaining social bonds within increasingly large human groups, laying the foundations for musical activity and subsequently promoting gene-culture co-evolution for musicality (Savage et al., 2021a).

Most previous research on the social bonding effects of music has focused on social bonds in general, rather than romantic relationships in particular. Indeed, group musicking seems to be the dominant mode cross-culturally (Shilton et al., 2023). Nevertheless, there are few examples of studies that have specifically examined the role of music in romantic pair-bonding (e.g., Sharon-David et al., 2019). As has been noted by Shultz and Dunbar (2007), human friendships greatly resemble the pair-bonds in other species in terms of both the time and cognitive resources invested. It has also been suggested (usually at the drinks reception after a seminar on the synchrony-bonding effect) that sexual intercourse is also a synchronized activity, and studies have indeed found that it generally leads to social bonding (Meltzer et al., 2017). Parallels have been drawn between the rhythmicity of music and sex (Safron, 2016), and therapeutic interventions for sexual dysfunction often emphasize the need for coordination between partners (Pierce, 2000). Although there are many activities that require some level of rhythmic coordination, there are few that require such tight, rhythmic coupling to achieve success, nor for which success is measured by the enjoyment of all participants (rather than in an external outcome such as locomotion in rowing). Sex also activates the same endorphin reward system as other social bonding behaviors (Jern et al., 2023). Indeed, among bonobos, a close relative of humans, sexual intercourse is regularly used to maintain social bonds and mitigate conflict within a group (Clay and Zuberbühler, 2012; Clay and de Waal, 2015). Bonobos also appear to have some rhythmic entrainment abilities (Large and Gray, 2015). This raises the possibility that both music-making and touch may function to maintain romantic bonds in a similar way to non-romantic bonds, but perhaps with different intensity.

#### 2.4.3 Functions of love

The evolutionary functions of music have already been discussed above, but what about love? Bode and Kushnick (2021) propose five different fitness functions of love: mate choice, courtship, sex, pair-bonding, and health.

Mate choice involves selecting and focusing attention on a preferred partner. This has efficiency benefits, but also opportunity costs and the risk of making an imperfect choice. It is therefore important to obtain high quality information about a potential mate before making a choice. Information about one’s suitability as a potential mate is often signaled through courtship behavior, which is also facilitated by romantic love (Buss and Schmitt, 2019). Courtship can involve both intersexual attraction and intrasexual competition when multiple individuals are competing for a mate (Bode and Kushnick, 2021). As discussed above, music and dance may be used as courtship behaviors as part of a mate choice process.

Romantic love is typically associated with increased frequency of sex and a greater likelihood of pregnancy (in the absence of contraception or infertility), which directly increases reproductive fitness. Although sexuality and reproduction play a secondary role in some forms of love (e.g., love among prepubescent children or at a higher age), love remains a primary motivation for sexual activity, and the decision to procreate is often preceded by a deep romantic commitment (Bode and Kushnick, 2021; Sorokowski et al., 2021). The expression of love is often cited as a reason to have sex, and sex also promotes bonding within a couple (Meltzer et al., 2017). This is not unique to humans, and there are other species with complex social structures that appear to have sex for social bonding purposes rather than purely for reproduction, notably bonobos and dolphins (Clay and Zuberbühler, 2012; Brennan et al., 2022; Demuru et al., 2022). It has been noted that behavioral synchrony may be an important factor in bonobo sexual interactions (Palagi et al., 2020). However, sex is also costly in terms of time and effort, it potentially exposes individuals to external threats as they must direct attention towards each other, and (at least in most contexts in contemporary society) cannot be performed in public. Other (synchronous) activities, such as music and dance, are less costly and involve less risk, therefore enable romantic partners to reinforce social bonds with fewer limitations.

Romantic love generally promotes pair-bonding (Fletcher et al., 2015). Human relationships fall within concentric circles, with the innermost circle usually containing only one or two other individuals (Dunbar, 2018). Couples in a romantic relationship typically experience a high level of bonding, which is effortful to maintain and may detract from relationships in one’s wider network. However, this high level of bondedness promotes greater resource sharing and cooperation in caregiving and childrearing, leading to the suggestion that love is an emotional system that supports pair-bonding (Campbell and Ellis, 2005). Furthermore, love involves fitness dependence, exemplified by commitment through jointly produced offspring and monogamy (Buss, 2018). However, the extent to which romantic love is necessarily tied to monogamy is fiercely debated (Diogo, 2019). In comparison, the bonding effects of music are not limited to the innermost friendship circle, and can be used for large-scale social bonding with groups of 100 or more (Weinstein et al., 2016). As such, both music and love may have a bonding function, but music may promote bonding in general, whereas love is more specific to pair-bonds.

Finally, love promotes the health and survival in both adults and their offspring (Fletcher et al., 2015), and this is likely to be the case for music. Romantic love has been associated with increased wellbeing (Esch and Stefano, 2005), which may be related to the general health benefits that accompany social connection (Holt-Lunstad et al., 2010; Schutter et al., 2022). Similarly, numerous studies have examined the links between music and well-being, particularly in relation to emotion regulation (Baltazar and Saarikallio, 2016), which may have an underlying social nature (Schäfer et al., 2020). Love, just like music, may be a ‘complex suite of adaptations and by-products’ (Bode and Kushnick, 2021). Although the health benefits provide some survival advantage, they are likely to be a by-product of more general social–emotional functions, whereas the effect of love in promoting reproduction and shared childrearing may be clearer adaptive functions. Similarly, the multiple abilities associated with musicality may have had different functions before being co-opted to support musical behavior (Savage et al., 2021b).

## 3 Bridging music and romantic love

In this chapter, we aim to combine theories of the evolutionary functions of music with social psychological theories of the stages and components of romantic love to propose a new model of the potential role of music in romantic relationships. We begin by introducing our theoretical framework, which includes both the stages of romantic relationship development (e.g., Mongeau and Henningsen, 2008) and the components of love according to the Triangular Theory of Love (Sternberg, 1986). In our proposed model, we summarize the evidence on how music can potentially enrich each component of the Triangular Theory of Love at different relationship stages.

### 3.1 Analytical framework

In our model of the role of music in romantic relationships, we draw on the components of the Triangular Theory of Love (Sternberg, 1986) and the different stages of relationships. While a brief introduction to the framework is provided here, a more detailed description of the theories used can be found in the Supplementary material, Sections 1–3.

Romantic love is a multi-faceted concept with a variety of interpretations, from ancient Greek concepts to cultural variations, making it difficult to provide a universal classification. Typical elements include strong emotional attachment combined with sexual desire and tenderness (Goode, 1959). The Triangular Theory of Love (Sternberg, 1986) is a widely used framework that identifies three components of love: intimacy, passion, and commitment. According to this theory, different types of relationships can be covered by the different weightings of these components, with romantic love including all three aspects. The theory has been very influential in research and has been empirically validated (e.g., Acker and Davis, 1992; Lemieux and Hale, 2002), including evidence of potential universality across cultures (Sorokowski et al., 2021).

The relative weight of these three components varies between couples and is also influenced by the stage of development of the relationship. During the late twentieth century, several stage models of romantic relationships have emerged addressing the stages from initial attraction to commitment declarations, including the Staircase Model (Knapp, 1978), Social Penetration Theory (Altman and Taylor, 1973), and Uncertainty Reduction Theory (Berger and Calabrese, 1975). However, the relationship effort extends beyond this point and requires different maintenance strategies to keep the relationship alive (Ogolsky et al., 2017). Overall, the different theories can be grouped into three overarching stages, which we use as structuring elements in our postulated model: the attraction phase, the relationship building phase, and the maintenance phase. While passion and intimacy in particular increase in the first stage, the second stage is dominated by intimacy and the third by commitment, with passion tending towards a slight decline (e.g., García, 1998; Wojciszke, 2002).

### 3.2 The MEL-model: framework for music, evolution, and love

**Figure 1:**
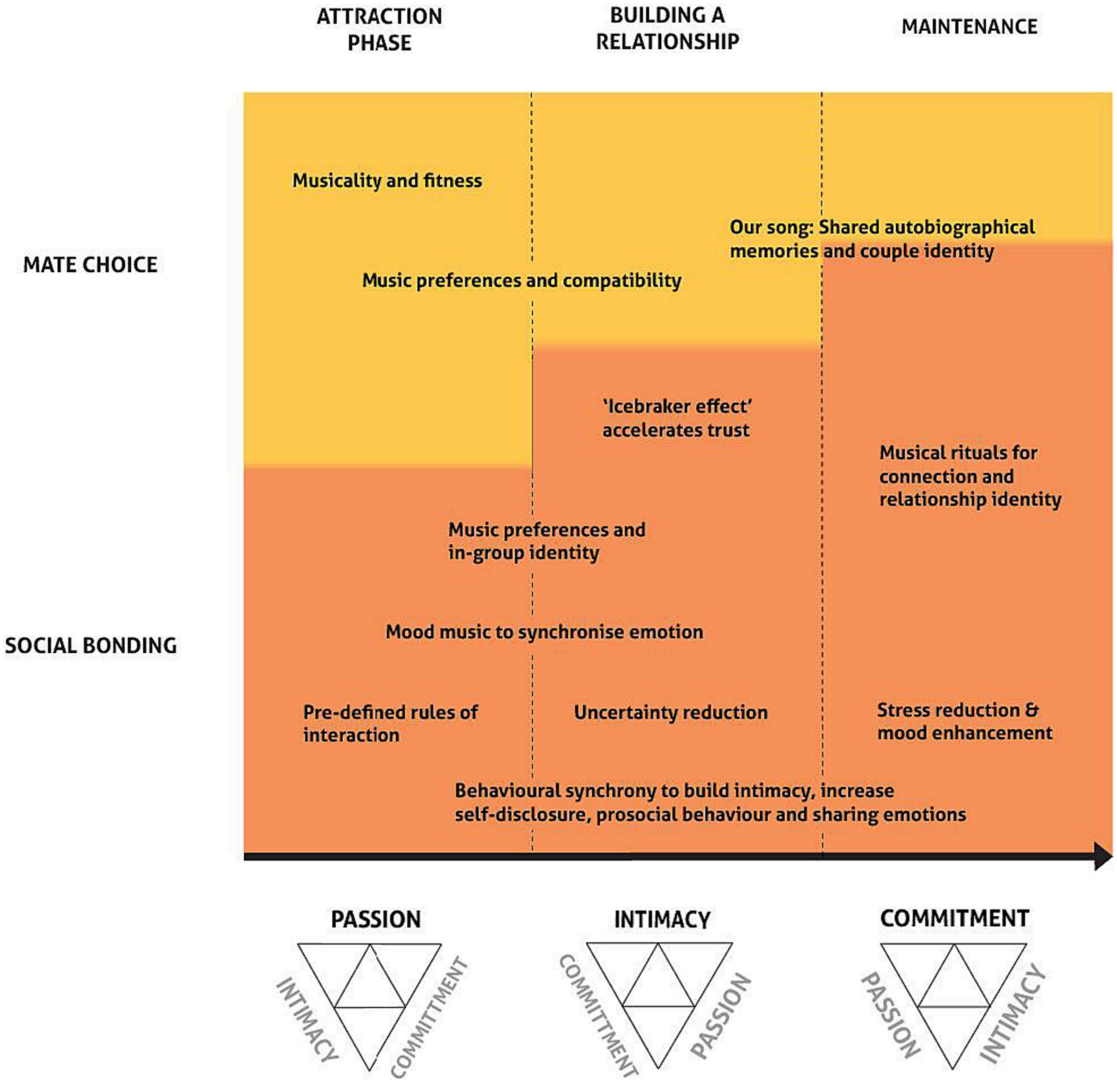
Visualization of the Music-Evolution-Love (MEL) Model. Relationship stages are arranged on the horizontal axis at the top, corresponding to the dominant aspect of Sternberg’s triangular model of love at the bottom. The vertical axis shows the relative importance of the social bonding and mate choice functions. Examples of specific uses of music are given within the diagram, based upon the existing research.

In the pursuit of a comprehensive synthesis encompassing relationship stages, the Triangle Theory of Love, evolutionary frameworks and the influence of music, our efforts have crystallized in the form of the Music-Evolution-Love (MEL) model, see Figure 1. This illustrative framework aims to combine theories of love and evolutionary functions of music to illustrate how music has the potential to enrich different components of love in different relationship stages. The MEL model, in its current form, is only intended to apply to human romantic relationships, as this is the focus of the current psychological literature on love and relationship stages, although future work could expand upon this. It should be noted that much of the work on music and social bonding has looked at social bonding in general, so Figure 1 is built on some speculation about how these general social bonding effects may apply in the use of music in romantic relationships in particular. We acknowledge that this model may look very different for other relationship types.

#### 3.2.1 Attraction phase—music in the first encounter

When considering a link between music and attraction, one might immediately think of the possible evolutionary function of music in terms of mate choice. In this chapter, we argue that the role of music relates to both mate choice and social bonding, while regarding the triangular theory, the components of passion and intimacy are emphasized (García, 1998; Wojciszke, 2002).

As mentioned above, there is evidence to suggest that music may serve as a means of signaling attractiveness, although some studies offer different perspectives on this issue (see also Mehr et al., 2021). Attractiveness, in the context of mate choice can either be an indicator of general fitness, or of compatibility; someone may appear attractive because they would be a good partner for anyone, or specifically for the perceiver based on personal factors.

At a general level, creativity is widely perceived as an attractive trait (e.g., Buss, 1989; Karamihalev, 2013). Furthermore, a relationship has been observed between artistic success and the number of sexual partners (e.g., Clegg et al., 2011; Lange and Euler, 2014), and individuals tend to be more creative after being primed with romantic motives (Griskevicius et al., 2006). The extent to which musicality is generally perceived as attractive remains somewhat controversial based on current research. In the study by Marin and Rathgeber (2022), people rated faces as more attractive and expressed higher dating desirability when the targets were presented as musicians, with this effect being particularly pronounced for women. Similarly, a study by Madison et al. (2018) demonstrated an association between the quality of musical improvisation and reported levels of mate value and mate preference. Another example of displaying attractiveness through music is dancing. This involves not only showing physical attractiveness (Weege et al., 2012) and strength (e.g., Hugill et al., 2009), but also the ability to coordinate with the music, and cooperate with a group or a dance partner (e.g., Hagen and Bryant, 2003). However there is also evidence against a general attractiveness of musicality. A study of over 10,000 twins found that there was no correlation between musical ability and performance and indicators of mating success such as number of sexual partners, sociosexuality or number of offspring (Mosing et al., 2015). Other studies have found that musicians are not perceived as generally more attractive, except to other musicians (Bongard et al., 2019), suggesting a preference for similarity or compatibility of interests, rather than musicianship as a marker of general fitness.

Music and dance also hold significance within the context of nightclubs, where a substantial amount of dating activity takes place. As observed on dance floors, the combination of music, dimmed lighting, and the cultural ambiance of nightclubs fosters heightened emotional experiences that facilitate connections between individuals. The sexually provocative lyrics commonly heard in dance music may make others appear more attractive (Carpentier et al., 2007). For instance, one study found that women were more likely to give their phone number to a man after listening to songs with romantic lyrics (Guéguen et al., 2010). Some people are acutely aware of how music can set the scene for romantic interactions, and will go to great effort to select the right music to support the level of passion they desire in that moment (DeNora, 2000, p. 113).^3^ The precise nature of the music may depend upon the interaction, as dance music tends to be fast and energetic to encourage movement (Mehr et al., 2018), while music associated with “intimate encounters” is more likely to be slower and altogether more sensual (DeNora, 2000, p. 116). Thus, musical affordances may be used to create a shared romantic space, supporting passion in a newly emerging relationship.

There is less direct evidence for music increasing intimacy and commitment in the attraction phase. In terms of intimacy, the musical activity of dancing may again be relevant, in this case not as a means of expressing attractiveness through dance movements, but as a shared musical experience. As described in section 2.4.2, movements that are synchronized to music and to a partner can increase feelings of connection and intimacy. Furthermore, a dance style has a structure and rules that allow for a safe and predictable social interaction (Kimmel, 2019), which could create a defined space for flirting (Kaminsky, 2011). Engagement in this shared space implies a mutual agreement without needing to make that agreement more explicit—sometimes referred to as ‘floating intentionality’ (Cross, 2014). Put more crudely, everyone on the dance floor has a culturally acquired understanding of where they are allowed to put their hands. This allows for more physical closeness than would be expected with a stranger in an unstructured interaction.

Another aspect that may be relevant to building intimacy with a previously unknown person is musical preferences. A study by Rentfrow and Gosling (2006) found that music was one of the most common topics of conversation between strangers trying to get to know each other. These preferences reveal a great deal about people’s personalities, values and social backgrounds, which can also be identified relatively accurately by the other person (see Boer et al., 2011, for a review). In particular, they provide insight into an individual’s arousal preferences and emotion regulation strategies (e.g., seeking high arousal with fast music; Cook et al., 2019), the desire for cognitive stimulation (reflected in a preference for complex music; Getz et al., 2014), and the inclination towards social interactions, openness and extraversion (e.g., frequent listening to party music; Vella and Mills, 2017). Thus, shared music preferences and similar personality traits and values derived from them can signal a high level of compatibility and foster a sense of intimacy and closeness with previously unknown individuals, consistent with the idea that similarity is important in courtship behavior (Bode and Kushnick, 2021) and contributes to social attraction (e.g., Luo, 2017). For example, shared musical preferences have been linked to friendship formation (Selfhout et al., 2009) and ingroup favoritism, such as stereotyping fans of different musical genres and perceiving those with similar musical tastes as ingroup members (Lonsdale and North, 2009). Music preferences are further being used in dating platforms such as Tinder, where users can showcase their personalities by selecting a specific song or, through Spotify integration, displaying their top artists based on listening patterns. This act of presenting one’s musical tastes serves as a powerful form of cultural communication, signaling to others “what kind of person” one is and potentially attracting like-minded individuals (Kang, 2023).

Whether music or musical preferences can also strengthen commitment in the early stages of a relationship is, to the best of our knowledge, not clear from previous evidence to date. Some relationships may even begin with a pre-existing commitment if, for example, a couple meet while taking a dance course together or through participation in a music ensemble. This experience would not be unique to musical contexts, as people commonly meet a partner through shared interest groups (see Byrne, 1997).

#### 3.2.2 Building a relationship—music for uncertainty reduction and bonding

The relationship-building phase involves getting to know each other better through intensified self-disclosure, reducing uncertainty and building deeper connections. Many of the mate choice processes of the attraction phase may still be relevant, as partners continue to choose each other, but general social bonding mechanisms become more important. Thus, passion still plays a role, but the intimacy component is the most important aspect to consider, with a marginal role of commitment.

Intimacy can be promoted by reducing uncertainty and gaining deeper insights into your partner’s personality, for example by discussing preferred music genres or musical activities. Musical taste is experienced as a meaningful way to express one’s identity and social positioning, as well as to find like-minded individuals (e.g., Schäfer and Sedlmeier, 2010; Shepherd and Sigg, 2015). It has been shown that the *Esthetic Self*, i.e., the taste in music and the visual arts, has a greater influence on identity self-concept than leisure activities such as hiking or playing video games (Fingerhut et al., 2021). Sharing musical preferences can thus help to communicate one’s own identity and identify a compatible and potential partner, while at the same time promoting the experience of intimacy. As a beneficial side-effect, it also has the potential to increase the passion component, as passion tends to increase significantly when there is a rapid increase in intimacy, such as through the exchange of more personal information (Baumeister and Bratslavsky, 1999).

Getting to know each other and reducing insecurities can be promoted not only by talking about music, but especially by engaging in musical activities together. As described in section 2.4.2, shared musical experiences can contribute to increased feelings of inclusion, connectedness, and positive affect (e.g., Harvey, 2017, p. 153; Weinstein et al., 2016). Collective singing was found to be more strongly associated with sociobiological bonding responses, as measured by the concentration of salivary oxytocin levels, a reduction in negative affect, and an increase in feelings of social connectedness compared to engaging a pleasant conversation (Kreutz, 2014; Bowling et al., 2022). Interestingly, improvised singing resulted in higher levels of plasma oxytocin, indicating increased social bonding, compared to pre-composed singing. This may be due to the interactive nature of improvisation, which requires more active listening, spontaneous communication and eye contact (Keeler et al., 2015). Furthermore, making music together (as opposed to simply listening to music or participating in less interactive musical activities) is associated with an increased pain threshold, a common proxy measurement indicating endorphin release (Dunbar et al., 2012). This process is associated with hedonic rewards (Berridge and Kringelbach, 2011) and that has also been observed in non-primate and primate bonding behavior (Machin and Dunbar, 2011).

As described earlier, these effects are largely driven by sensorimotor synchronization and coordination, the predictability of these actions and their activating reward networks (Clayton et al., 2020; Hesp et al., 2021; Bamford, 2022). This result is consistent with the expansion of the self theory (Aron and Tomlinson, 2018): individuals seek to expand their sense of self and experience an inclusion of the other within themselves, something that could be facilitated by synchronized actions.

However, synchrony seems to depend on the social context and mutual liking. A study by Miles et al. (2010) showed that participants were less likely to synchronize their movements with a partner who had kept them waiting for 15 min at the beginning of the study, thus engaging in negative behavior. The authors conclude that the presence or absence of synchrony could be subconsciously used to establish or terminate social relationships. Applied to romantic relationships, it could be speculated that (musically) experienced synchrony or lack of synchrony in the early stages of dating could be an indicator of mutual positive attitudes and whether further intensification of the relationship is possible.

Apart from promoting bonding in general, making music together also helps to make bonding happen *more quickly*, referred to as the ‘icebreaker effect’ in a study by Pearce et al. (2015). In this quasi-experimental study, singing groups were compared with non-singing groups (craft or creative writing groups) across three measurement points. While all groups reported similar levels of closeness after 7 months, the speed of bonding differed considerably. Singing groups had higher initial closeness levels than non-singing groups. This finding was attributed to the shared pursuit of a common goal (making music) and experienced synchrony, which accelerates trust and liking even with limited information about group members. Although shared interests in general are seen as a good way to meet people (Byrne, 1997), music and dance may have additional benefits because of the synchrony involved.

Of course, this result does not translate directly into the development of romantic relationships. However, it could be argued that even in a dyadic setting, shared musical activities could facilitate quicker bonding compared to ordinary small talk or other activities. Thus, the “icebreaker effect” may allow individuals to bypass superficial and rule-bound communication, leading to faster trust, self-disclosure and deeper exchange. As a result, this accelerated process may help partners to decide more quickly whether or not to continue the relationship and to gain clarity about the suitability of their potential partner. In the language of relationship stage models, this would include music contributing to a faster reduction of uncertainty (Berger and Calabrese, 1975), social penetration (Altman and Taylor, 1973) and the process of revealing personal information and thoughts that deepen the bond.

In addition to these aspects, which are more related to the strengthening of intimacy, music making can further help to increase feelings of commitment. For example, engaging in synchronized action, such as shared musical activities, involves a certain amount of shared intentionality, as a common goal is being pursued, which leads to more cooperative and prosocial behavior towards other performers (Reddish et al., 2013). Furthermore, it also involves shared attention and shared success (Wolf et al., 2016), which together with experiences of motor synchrony are associated with an increased sense of commitment, belonging and responsibility towards the other performers (Kokal et al., 2011). These effects may arise from any coordinated action, not just musicking, but temporal synchrony appears to be important to the effects (Rennung and Göritz, 2016; Vicaria and Dickens, 2016; Mogan et al., 2017), and there are few activities that require such precise temporal synchrony as music. Therefore, when applied to romantic relationships, it could be expected that joint musical activities in the relationship-building phase could promote a sense of commitment, mutual belonging, responsibility and affiliation, possibly even more quickly than other activities, in the sense of a further ‘icebreaker’ effect.

#### 3.2.3 Maintaining a relationship—music as tool for relationship enhancement

To foster and sustain a satisfying relationship, a range of maintenance behaviors comes into play, driven by the dual motivations of threat reduction and relationship enhancement (Ogolsky et al., 2017). At this stage of the relationship, the role of music in mate choice is rather secondary, while (re)bonding is of primary importance. As defined by Wojciszke (2002), in the later phase he labeled as “companionate love,” relationships prioritize commitment and intimacy over time, with a reduced emphasis on passion.

One of the most salient relationship maintenance strategies to promote commitment involves sharing leisure time and activities. In this context, music is often cited in inventories of maintenance strategies (e.g., Girme et al., 2013). In a study by Harwood and Wallace (2021), engaging in dyadic musical activities (e.g., sharing music, listening together, singing), was associated with higher commitment, mediated by interpersonal coordination and self-disclosure, even when controlling for other shared dyadic activities (Harwood and Wallace, 2021). Notably, the effects were observed only in dyadic musical activities shared with partners, as opposed to structured musical group activities. This distinction may imply different musical bonding mechanisms tailored to friendships and romantic partnerships.

Another tool for strengthening commitment is the phenomenon of “couple-defining songs.” Specific songs associated with a relationship are not uncommon. For example, in a study by Harris et al. (2020), 60% of participants reported having such songs. The choice of a particular song typically occurs early in the relationship building phase with the primary motive of creating a shared couple identity and a sense of “we,” which can be seen as a strong sign of commitment, just as music can be used to reinforce an individual identity (Nora, 2019). At the same time, the shared conscious or unconscious choice of a common song can be a sign of compatibility and promote the development of the relationship by recalling memories from the attraction phase; “our song” may remind you why you chose this partner in the first place. Autobiographical memories associated with music are often accompanied by positive emotion (Jakubowski and Ghosh, 2021). The association of the shared song with positive relationship memories and feelings of nostalgia—and thus shared autobiographical memories (Alea and Bluck, 2007)—has the potential to restore intimacy, passion and commitment by listening to the song, even at later points in the relationship and as a relationship maintenance strategy.

Another aspect that quickly comes to mind when thinking about commitment and creating stability in romantic relationships is ritual. Interpersonal rituals, as defined by Wolin and Bennett (1984), are characterized by their ability to transcend daily routines, focus on family identity, foster communication and evoke intense emotions. Couples’ rituals tend to be distinctive and personalized, contributing to the cultivation of a unique shared culture and thereby fostering enduring relational bonds (Pearson et al., 2010). There are many forms of ritual in couple relationships, including shared time, daily tasks, communicative goals, or spiritual activities (Bruess and Pearson, 1997). Another example are joint musical activities such as making or listening to music, frequently reported by the participating couples in a study by Campbell et al. (2011). Charles Darwin himself noted gaining great pleasure from hearing his wife Emma practicing the piano, and this ritual seemed to be a comfort to him in his old age (Bannan, 2020). Drawing parallels with family dynamics, research suggests that musical rituals are associated with improved family cohesion, well-being, and protection from crisis (Wolin and Bennett, 1984; Boer and Abubakar, 2014), suggesting a similar potential impact within romantic couples.

Again, synchrony in music may also have the potential to increase commitment in romantic relationships. For example, previous research has shown that individuals tend to help those with whom they have synchronized and display prosocial and cooperative behavior towards them (e.g., Wiltermuth and Heath, 2009; Valdesolo et al., 2010; Reddish et al., 2013). In the context of couples, this suggests that musical activities during the maintenance phase could not only foster closeness and passion, but also strengthen mutual commitment.

With regard to the positive effects of music on intimacy and passion, the aspects discussed above may also apply to the maintenance phase. For example, experiences of synchrony in music making or dancing may increase liking and feelings of self-other overlap in times of reduced intimacy (Hove and Risen, 2009; Lang et al., 2017). Notably, a study demonstrated how synchrony enhances intimacy among established heterosexual couples and even elevates sexual desire, suggesting that musical synchrony could heighten not just intimacy but also passion in couples (Birnbaum et al., 2016). This effect could counteract commonly cited reasons for divorce, such as “growing apart” and “not being able to talk to each other” (Hawkins et al., 2012), given the potential of music making to promote partner responsiveness, which in turn enhances intimacy (Reis et al., 2004).

There is also some evidence from couples therapy, particularly in the context of couples where one partner is hospitalized, and from marriage counseling. Overall, these studies suggest that engaging in shared musical activities, such as playing or listening to music together, is associated with both positive emotions and increased feelings of closeness in couples (Hinman, 2010; Stedje et al., 2023). Music may also serve as an honest signal to communicate emotional state (Harvey, 2017, p. 133), so it could have the potential to improve couple interaction by refining communication skills (Botello and Krout, 2008). For example, collaborative improvisation can serve as a means of expressing current feelings about different relationship aspects and individual needs (e.g., the need for calm or excitement), and then working together to address those needs (Duba and Roseman, 2012; Palmer, 2018; Fraenkel, 2020).

A major source of relationship problems that should be addressed by maintenance strategies is stress. This is an issue across mammal species, with increased stress being associated with reduced fertility, which serves as an important selection pressure for stress reduction behaviors (Dunbar and Shultz, 2021). According to stress spillover theory (Randall and Bodenmann, 2009), external stressors such as work, finances, or problems with friends and family can trigger internal stress, by leading to reduced quality time, communication problems, and increased expression of problematic personality traits in partners (e.g., anxiety, dominance, rigidity). In addition to these aspects, which can best be attributed to the intimacy component, stress is also associated with less sexual desire, which particularly affects the passion component (Bodenmann et al., 2010).

So how can music contribute to maintaining relationships during stressful times? One potentially important aspect is using music for emotional regulation and stress reduction. This relates to the social bonding hypothesis, which suggests that musicking alleviates the stress of social living, and subsequently reinforces social bonds with those who help to reduce our stress (Dunbar, 2012). Indeed, when faced with stress, individuals tend to increase their music consumption (Getz et al., 2014), and, when chosen appropriately, music can effectively contribute to personal stress reduction and mood regulation (Linnemann et al., 2015; Baltazar and Saarikallio, 2016; Baltazar et al., 2019). This emotional regulation can occur on an individual level, mitigating stress spillover effects within the relationship through its stress-relieving influence; or it can occur together as a couple. It was shown that listening to music with a close friend or partner tends to evoke more intense emotions, potentially leading to a stronger stress-reducing impact (Liljeström et al., 2013).

The emotion-regulating function of music may also play a role in conflict management, as suggested in a qualitative study by Smith and Martin (2020). Participants reported that listening to music alone after an argument can be helpful in calming down and thinking more clearly, while listening to music together or making music after an argument can restore intimacy. To our knowledge, there is only one study that has experimentally investigated joint music listening in couples. In this study music listening affected bio-psychological stress markers in both partners, with certain responses being gender-specific. Furthermore, the impact of this shared musical experience was dependent on individual music preferences and their arousal preferences (Wuttke-Linnemann et al., 2019), something to consider when selecting appropriate music for shared listening sessions.

## 4 Discussion

Through this paper we have surveyed the previous literature on the evolution of music and love, in connection with current psychological theories of romantic relationships. We must acknowledge that much of the science remains unsettled, and the role of this paper is not to settle it but rather to identify and propose future research questions.

The evolution of music has attracted increased attention recently, but the most prominent theories have largely ignored the possible role of sexual selection (although see Ravignani et al., 2017; Bannan et al., 2022; Leongómez et al., 2022; Marin and Rathgeber, 2022). The evidence may not be there at present, but the absence of evidence is not the evidence of absence. These previous accounts have centered the possible role of music in social bonding, but only in a general sense and not specifically in the context of romantic relationships. In this paper we have attempted to address both of these functions, suggesting focused investigation into the role of music in romantic relationships, either in attraction (sexual selection) or connection (social bonding). It is possible that both functions may exist, perhaps with different relevant importance depending upon the stage of relationship, but also upon cultural context. If these functions are operating in contemporary society, this also does not necessarily mean that they have served as selection pressures for musicality throughout human history, as certain aspects of musicality may have been co-opted for these purposes, as will be discussed.

### 4.1 Evolutionary mismatch

Both music and love may now serve very different functions, and be expressed in very different ways, than they were for our early ancestors, which may indicate an evolutionary mismatch (Li et al., 2018). Given the advancement of communication technologies, humans now have access to a wider variety of potential mates than ever before, do not require close proximity, are less integrated into a local community, and may move around more (Goetz et al., 2019). Romantic love is also expected to last longer as life expectancy has increased (although high divorce rates in many countries may suggest that this expectation is rarely met), and has also become increasingly decoupled from childbirth with the advent of contraception (Bode and Kushnick, 2021). Many of the features of romantic love that are considered norms in WEIRD societies (Henrich et al., 2010) may be the product of a culture adapted to agricultural living, which differs greatly from the conditions for most of human evolution (Ryan and Jetha, 2010, p. 9). Western notions of love strongly emphasize the romantic, but there is great diversity in the way people form relationships across different cultures, including love marriages, arranged marriages, walking marriages, polygyny, polyandry, and serial monogamy. All these factors may change the way love is developed and expressed.

Similarly, the role of music may be very different in modern society than it was for much of our evolutionary history. For one, music listening is clearly of great importance to contemporary humans, particularly in sharing musical preferences with potential friends and partners. However, music *listening* would have been unheard of before the advent of recording technologies. Music, for most of our evolutionary history, was something that was *done.* For instance, even in most contemporary societies, group singing is far more common than solo singing (Shilton et al., 2023), which would not appear to be the case if we were only to study Western pop. Therefore, the use of musical taste as a means of assessing partner compatibility is likely a more recent cultural innovation and is not relevant to discussions of evolutionary history. However, shared cultural knowledge of music and dance may be an important marker of group identity that could have been used to assess compatibility of potential partners long before sharing spotify playlists on Tinder was possible. The technologies may change, but perhaps the functions remain the same. Human technology has also enabled an increasing range of entertainment opportunities, and social situations (e.g., a music festival) consisting of thousands of people, where for much of human history any individual would be unlikely to interact with more than 150. Given the present range of cultural diversity in romantic relationships as well as in musical traditions, we may expect that the use of music in romantic relationships may differ between cultures.

### 4.2 The order of things

Throughout this paper we have entertained both the sexual selection through mate choice, and social bonding hypotheses of the evolution of musicality. Both of these suggest that relationships (romantic or otherwise) are foundational to the evolution of musicality, whether it is for attracting a mate or maintaining a relationship. However, it may be reasonable to ask which function came first. We suggest that music for attraction may have emerged after music for bonding, although this is difficult to test empirically. In terms of the abilities required, the social bonding effects of music seem to rely mostly upon rhythmic synchronization (Hove and Risen, 2009), and so this function may even predate music as we know it, while the sexual selection function could require a more fully-formed musicality. Many sex differences may be cultural, so sexual selection for more advanced musicality may have begun after basic musical capacity had already evolved (Savage et al., 2021b). If musicality became important for either group bonding or parent-infant bonding, then a display of musicality may have become an honest signal of one’s social status or parental ability, which could then be sexually selected for. On the other hand, the use of music to signal personality/preferences may be an exaptation, as sharing personal playlists must have emerged after musicality and the subsequent cultural evolution of music. Otherwise, there would have been no diversity in music to signal preferences.

Given the interrelated nature of music and love, these two phenomena may have co-evolved. Romantic relationships require communication, and early music-like communication may have been vital in communicating basic emotional states as a form of self-disclosure during the relationship building phase in early humans. This may also be reflected in the similar hormonal systems engaged by music and love.

Some have suggested that musicality may simply be a byproduct of cognitive abilities that developed for language, a kind of ‘auditory cheesecake’ (Pinker, 2003), and that language may be a better bonding mechanism than music (Mehr et al., 2021). However, synchronous chorusing was likely an intermediate step towards language (Merker, 1999; Dunbar, 2003). Synchronized singing would lead to greater fidelity of transmission, while asynchronous speaking may lead to more fragmentation and the development of dialects (Cohen and Haun, 2013). Even if language were to come first, it is worth considering that mate choice could still have a role; cheesecake itself may provide no survival advantage, but cheesecake is delicious, and the people who can make cheesecake may be more likely to attract a mate.

It is quite likely these adaptationist and byproduct theories can be reconciled through gene-culture coevolution, consistent with the approach of Savage et al. (2021b). Aspects of musicality may have begun as byproducts of some other ability, only to become exaptations that serve musical behavior. Cultural innovation, however, can shape the environment, and if more musical humans had some advantage over less musical humans then adaptations specifically for musicality may arise (Patel, 2018). Such survival advantage could be because musicality helps individuals to gain social support, increasing their own survivability (Savage et al., 2021b), or because groups that can make music together have an advantage (multilevel selection; see Okasha, 2008). However, musicality may also have made people better parents (Leongómez et al., 2022), which means it could also serve as a signal of parental fitness. Alternatively, if joint musicking has a role in relationship maintenance, as a special case of social bonding, then this may have survival benefits for individuals who are better able to bond with their reproductive partners through enhancing romantic love.

### 4.3 Predictability in music and love

Predictive processing could serve as a unifying lens. A potential mate is a highly unpredictable stimulus. Similarity improves predictability, leading to an initial sense of rapport. Successful coordination through music-dance may be an honest signal of shared priors; sharing the same music/dance knowledge may indicate similarity in terms of interests or cultural background in a way that is very difficult to fake (Podlipniak, 2023). Sexually selected traits are often also hedonically marked (Rosenthal and Ryan, 2022), which could arise from the processing fluency effect of prediction fulfillment that may underpin the social bonding effects of synchrony (Bamford, 2022). Sustained interaction (through the building phase) increases predictability, as we learn more about the partner (increased passion and intimacy). Synchronized activity (i.e., music-dance) is a useful tool at this stage, because it makes the other more predictable relative to the self (Vesper et al., 2011; Bamford, 2022; Kret and Akyüz, 2022; Ravreby et al., 2022). A relationship becomes increasingly stable as a partner becomes more predictable. Social embodied predictions are made relative to oneself (Clark, 2015), and the more predictable the other is, the more one incorporates them into one’s model of oneself, which may relate to the self-expansion model of love (Aron and Tomlinson, 2018). Commitment may also be dependent upon high predictability—we know they will reciprocate. However, if a partner is too predictable then there is no passion, as there is no room for changes in intimacy. This is consistent with findings that there is an optimum level of complexity in interpersonal coordination (Ravreby et al., 2022). Within a predictive processing framework, agents avoid environments that are too predictable, because they are motivated to seek epistemic value, and in this sense a relationship, or a synchronized interaction, which is too predictable may resemble the dark room problem (Friston et al., 2012). Music overcomes this through striking a balance between predictability and surprise (Stupacher et al., 2022), potentially creating a shared space for optimum relationship building. In this way, a musical interaction may be a microcosm of a relationship.

This framework may predict that musicking would vary in its predictability according to function. The nature of music is not only shaped by selection pressures, but also by esthetic constraints that are inevitable consequences of human perceptual systems and psychophysics (Ravignani et al., 2017), and skilled musicians are able to play with sound to suit human ears. Music for connection may tend to be more predictable, to encourage synchrony, while music for attraction could be more complex, to signal the technical and creative skill of the performer. There is some preliminary evidence for the role of complex music in mate attraction (Charlton et al., 2012; Marin et al., 2017), but further testing is needed to draw more definitive conclusions. Existing research categorizing music by its function, specifically love songs and dance songs (Mehr et al., 2018), may provide a basis for further investigation.

### 4.4 Specificity of music

An important consideration for our model is the uniqueness of music’s role for relationships. As stated in the introduction, this review does not claim that music is necessarily superior to all other activities in fostering and maintaining relationships. While some of the effects discussed can be achieved equally well through alternative activities, others may be better facilitated through music. This is something our future research questions would aim to address.

For example, sports rituals or conversations about favorite books could replace the use of music for rituals or as a means of expressing personality. However, when it comes to the process of getting to know each other and assessing compatibility, music has some distinct advantages. It allows individuals to interact simultaneously without the need for turn-taking, thus fostering collaborative interactions without excessive cognitive effort (see the synchrony-bonding effect, e.g., Hove and Risen, 2009; Pearce et al., 2015; Bamford, 2022). In initial encounters, discussing musical preferences may be more accessible than directly expressing attitudes and values (e.g., Rentfrow and Gosling, 2006). In addition, engaging in activities such as playing music or dancing during the early stages of relationship building provides structured yet intimate interactions that may not otherwise be achieved, i.e., there are few circumstances other than partner dance in which it would be socially acceptable to hold a stranger’s hand for a prolonged period of time.

In terms of coordination and synchronization, although activities such as playing sports together serve this purpose, music and dance are unique in that they require very precise temporal coordination that extends over minutes or even hours. Sexual intimacy may also serve a similar social bonding function, although it comes with more risks and limitations (e.g., energy costs, risk of sexually transmitted disease, and the need for appropriate social context; Da Ros and Da Silva Schmitt, 2008; Frappier et al., 2013). Collaborative music-making further not only promotes coordination, but also results in the creation of something novel and potentially beautiful, involving shared goals and joint attention.

Another notable characteristic of music is its strong emotional impact (e.g., Juslin and Laukka, 2004). Music serves as a tool for individual (and potentially dyadic) emotion regulation, triggering self-regulatory processes (e.g., Liljeström et al., 2013; Getz et al., 2014; Baltazar et al., 2019). In this context, music takes on a deeply personal role, functioning as an affective extension of one’s identity. This characteristic likely contributes to music’s efficacy as a tool for navigating personal relationships, potentially making it more suitable for relationship enhancement than activities with comparatively lower emotional impact.

These examples illustrate the potentially unique role of musical components in fostering certain aspects of relationships. There are likely many mediating factors, depending upon individual personality and cultural context. As discussed previously, the human social environment has changed dramatically since the first humans sang, and so other behaviors may have come to serve roles that used to be filled by music. However, whether music outperforms other activities in achieving these effects, and whether these results are universal or dependent on specific personality traits or leisure preferences, remain questions that require empirical investigation.

### 4.5 Testing and applying the model

In this paper, we developed a model illustrating how music might enhance intimacy, passion, and commitment across relationship stages, from initial attraction to the maintenance of romantic relationships. While certain facets of the model draw directly from existing literature on romantic love, others are extrapolated from broader insights into social bonding. Thus, empirical testing of the various components of the model is essential, and we outline a number of potential research questions in the following section.

An important consideration involves the components and phases of the relationship. In our literature review, we found evidence that music, especially through coordinated and synchronized movements, can promote intimacy, which has also been shown in few studies with couples. However, the empirical basis for passion and commitment is less clear. Therefore, investigating the effects of different musical activities on these dimensions could significantly enrich our model.

We further proposed that the role of music and beneficial musical activities would change with relationship phases. The extent to which musical activities actually have a positive effect on the progression from one phase to the next could be clarified with various questions, for example:

- How prevalent is music in the attraction phase and how many couples bond over shared musical activities or musical preferences?
- Can musical preferences and activities contribute to getting to know someone faster and to recognizing compatibility?
- Do shared musical activities, for example dyadic improvisation or dance, reveal as much or more about a person or dyadic compatibility than, for example, small talk or a photo on tinder?
- Are musically compatible couples (i.e., in the sense of musical taste, music perception abilities, rhythmical abilities) happier?
- How well can musical compatibility predict whether relationships work, compared to similarity in personality or values?
- Can the music of attraction and the music of connection be distinguished on the basis of musical features? In particular in regards to the use of predictable structures.

A further aspect to consider is directionality. In our model, we propose that engaging in musical activities can have a positive impact on relationship development, fostering factors such as increased intimacy. In addition, we recognize that musical preferences and shared experiences may provide insights into each other, potentially serving as a predictive tool for assessing compatibility. Moreover, within established couples, musical interactions, such as coordination or roles in musical improvisation, may provide clues to communication quality and negotiation skills. This suggests that musical interactions may have potential as a diagnostic tool for addressing problems in romantic relationships.

Once the model has been further empirically validated, it could also be used to support couples at different relationship stages. For example, synchrony may be important for therapy and conflict resolution (Paxton and Dale, 2013; Nyman-Salonen et al., 2021). A music-based couples therapy could utilize musical synchronicity and coordination exercises to promote mutual prosocial behavior and commitment, while improvisation exercises could be used to promote effective communication and increase mutual attention and communication quality.

### 4.6 Expanding the model

As we delve deeper into the complex interplay between music and romantic relationships, it becomes essential to explore the nuances and limitations of the proposed model. In what follows, we will present questions about the applicability, uniqueness and specific dynamics of music in relationships that could be included in future research.

Some initial questions that may arise are: Who does the model work for? Can music universally improve romantic relationships, or does its effect depend on individual and relationship-specific factors? Are certain personality traits or experiences, such as exposure to nursery rhymes in childhood, likely to interact with musical activities and enhance their effects, particularly in promoting intimacy? Are there individual differences in the relevance of music for one’s identity and emotional life and do these differences determine the relevance of music for love? Furthermore, could music, despite its capacity for harmony, potentially create tension in relationships? If so, how might different musical choices reduce or increase such tensions?

Similarly, it’s worth considering whether romantic relationships have a distinct role. The present work intended to differentiate the impact of music within the realm of romantic love from its broader influence on social bonding. In some ways, friends and romantic relationships are very similar (Dunbar, 2018), and music may enhance love in all its forms. The process of increasing intimacy and self-other overlap seen in romantic relationships may be no different to general group bonding effects with friends, while there may be differences in the use of music to enhance passion and commitment, which play a subordinate role in non-romantic friendships.

Another aspect regards the operationalisation of love. Love encompasses a range of experiences, including emotional connection, empathy, compassion and a deep sense of affection. These facets of love are inherently subjective and difficult to quantify objectively. In addition, perceptions of love can vary widely between individuals and across cultures. While the Triangle Theory used in this study provides a framework that accounts for some dimensions of love that evolve over time, it does not include elements such as relationship satisfaction, different stages of love progression (e.g., arranged marriages evolving from commitment to intimacy and passion), or variations in the quality of love.

Furthermore, our model aimed to include stages of relationship formation and maintenance, but not dissolution. One avenue for future research is to explore how music can serve as a coping mechanism, regulating emotions, reducing stress and addressing feelings of loneliness during relationship dissolution.

Finally, we may consider how the MEL model could apply to non-human animals. There is comparatively little research on relationship stages in other animals, but this could be addressed in future ethology studies. However, there is anecdotal evidence, at least in Gibbons, that duetting behavior may be indicative of general relationship quality (M. Spierings, personal communication, 26 August 2023). Music-like behavior for mate attraction has been well documented across many taxa (Rose et al., 2022), but it may be worth considering how the music-like behavior of non-human primates, in particular, may be used for the formation and maintenance of relationships.

## 5 Conclusion

Throughout this paper we have discussed the role of music in romantic relationships, from an evolutionary perspective. Through a survey of the literature, we propose a model integrating sexual selection and social bonding hypotheses regarding the evolution of music, considering how each of these functions may operate at different stages of a romantic relationship. This is a significant expansion of the social bonding hypothesis, which hitherto has been applied to relationships in general, but mostly not to romantic relationships in particular. It also calls for greater consideration of the possible role of sexual selection for human musicality. We suggest that music and musicality may serve multiple functions in romantic relationships, some of which may be recent exaptations. Music for attraction may be used either as a cue to general fitness or compatibility, while music for connection may utilize the general social bonding effects of music as a means to promote interpersonal synchrony and liking. The attraction functions are likely more important in the early stages of any relationship, and may help to build passion, while the connection functions may help to build intimacy and commitment in later phases. Both these functions may leverage predictive processing, with the optimum level of predictability being determined by the function—higher predictability facilitates connection, while higher complexity may signal greater creativity. As a social bonding mechanism, musicking may be intimately related to the biological functions of love, engaging many of the same hormonal/emotional systems and potentially sharing an evolutionary history—without love, there may be no music. Although built upon prior literature, more research is needed to fully understand the interplay of love songs and serenades.

## Data availability statement

The original contributions presented in the study are included in the article/Supplementary material, further inquiries can be directed to the corresponding author.

## Author contributions

JB: Conceptualization, Project administration, Writing – original draft, Writing – review & editing. JV: Conceptualization, Writing – original draft, Writing – review & editing. MH: Visualization, Writing – original draft, Writing – review & editing. SS: Funding acquisition, Resources, Writing – original draft, Writing – review & editing, Conceptualization.

## Footnotes

^1^It is also perhaps significant that many musical terms may also have their origins in sexual inuendo. For instance, “rock’n’roll” began as American slang referring to sexual intercourse, and “jazz” may be derived from the Ki-Kongo word for ejaculation (Ventura, 1985).

^2^However, an interesting expansion of our proposed model could look at the role of social context and the use of music in intrasexual rivalry. Some examples could perhaps be found in rap battles or break dance competitions.

^3^Meanwhile, selecting the wrong music for a romantic encounter can end in disaster. e.g., https://www.reddit.com/r/tifu/comments/x35iu6/tifu_my_20f_girlfriend_of_two_years_told_me_the/. Although this story is likely not truthful, the joke only works because people seem to have an intuitive understanding of what romantic music should sound like.
