# Supplementary material for: Love songs and serenades: a theoretical review of music and romantic relationships
Source: Front Psychol. 2024 Feb 14;15:1302548. doi: 10.3389/fpsyg.2024.1302548 (PMC10899422; doi:10.3389/fpsyg.2024.1302548)
Supplement: Supplementary file 1 [file Data_Sheet_1.pdf]

SUPPLEMENTARY MATERIAL: Love Theories and Relationship Stages

The Multifaceted Nature of Romantic Love

Romantic love is so often written, spoken, filmed, and sung about, everyone has an intuitive understanding of what love is, even though there are actually many different types and definitions. These interpretations encompass a wide spectrum, spanning from ancient Greek notions like 'agape' or 'eros' (Aristotle, 350 C.E.; Gartner, 2017; Lewis, 2012) to cultural constructs such as the Confucian principle of 'ren,' the Sanskrit term 'kama' that encapsulates pleasure, delight, and longing in Hinduism, Buddhism, and Jainism (Monier-Williams et al., 1963, p. 271). Just as there are different terms, there are numerous definitions of romantic love that coexist, making the task of defining it universally difficult. However, some common elements include strong emotional attachment combined with sexual desire and tenderness (Goode, 1959), the inclusion of aspects such as passion, intimacy, and commitment (Sternberg, 1986), the longing for an enduring bond with a specific individual (Hatfield & Rapson, 1993), or the inhibition of attention to other partners accompanied by distinct emotional and physiological markers (Fletcher et al., 2015).

There are various theories that aim to explore love's origins e.g., evolutionary theory of love (L. Campbell & Ellis, 2005; Fletcher et al., 2015), attachment systems (e.g., attachment theory, Ainsworth, 1989; Bowlby, 1999), or components (e.g., colour wheel theory of love, Lee, 1973). In the present theoretical work, we draw primarily on the widely recognized Triangular Theory of Love (Sternberg, 1986), complemented by phase models that outline the development of relationships over time (e.g., Mongeau & Henningsen, 2008).

Triangular Theory of Love

According to the Triangular Theory of Love (Sternberg, 1986), there are three main components of love, representing the corners of a triangle: Intimacy, passion and commitment. Depending on how strongly each component is weighted, different types of human affection can be distinguished, ranging from *non-love,* in which all components are absent, to *liking*, in which there is much intimacy but little passion and commitment, to *consummate love*, in which all three components are strongly expressed.

The first component, *intimacy*, represents the emotional or 'warm' component of love. Common definitions of intimacy include mutual interaction, communication and self-disclosure, deep affective and cognitive awareness and expressiveness, willingness to show vulnerability, and strong closeness to each other (Moss & Schwebel, 1993; Sternberg, 1986). Intimacy is considered one of the most essential human needs in attachment theory (e.g., Bowlby, 1999), and previous research has shown that the level of intimacy experienced in a relationship or other forms of social connection is a predictor not only of relationship satisfaction and functioning (e.g., Goodman, 1999; Patrick et al., 2007), but also of physical health (e.g., Hale et al., 2005) and psychological well-being (e.g., Sneed et al., 2012).

Intimacy is closely tied to self-expansion theory; those who actively pursue intimacy often experience increased self-expansion with their partners (Harasymchuk et al., 2020).

Passion, as the second component, captures the motivational or 'hot' component of love (Sternberg, 1986). Passion consists of cognitive aspects such as persistent thoughts about one's partner, affective aspects such as sexual attraction and strong emotions, and behavioural aspects such as seeking physical closeness (Hatfield & Sprecher, 1986). Passion is often used interchangeably with romantic love, and it contrasts with companionate love (Hatfield et al., 2012). However, as described above, passionate love involves more than just sexual desire and cannot be sustained solely by it (Carswell & Impett, 2021). The constructs of passion and intimacy are strongly correlated and interdependent (Rubin & Campbell, 2012). For example, Baumeister and Bratslavsky (1999) postulate that passion is particularly intense when intimacy increases rapidly, such as after spending time apart or when new activities are shared. Similarly, according to self-expansion theory (Aron & Tomlinson, 2018), passion decreases over time as boundaries of closeness and self-expansion are reached, but increases again when new and 'expanding' experiences are shared with the partner. Therefore, activities that promote intimacy might be expected to indirectly lead to an increase in passion.

Finally, *commitment* represents the cognitive or 'cold' component of love, that is, the decision and dedication to be in love in the short-term and to maintain a relationship in the long term. In empirical investigations, commitment has been shown to be the strongest and most consistent predictor of relationship satisfaction (Acker & Davis, 1992; Kochar & Sharma, 2015). It is further associated with relationship-maintaining behaviours, such as adaptive social comparisons, devaluation of threatening alternatives, willingness to make sacrifices for the partner, and a tendency to accommodate rather than retaliate in response to negative partner behaviour (Wieselquist et al., 1999). These attitudes also promote reciprocal relationship-maintaining behaviours in the partners that contribute to the stability of the relationship (Rusbult & Buunk, 1993).

The triangle theory of love has been criticised, and there are alternative theories that aim to portray love in a more complex way. Nevertheless, it has been highly influential and has served as a basic framework for other theories of romantic love. It has been empirically validated (e.g., Acker & Davis, 1992; Lemieux & Hale, 2002) and has demonstrated potential universality across cultures (Sorokowski et al., 2021). Given its importance and empirical support, we consider it essential to include it as a foundational theory in this article.

Romantic Relationship Stages

When studying romantic relationships, it's important to recognise the different processes taking place before individuals identify as *friends* or a *couple*. In order to delineate these different stages of relationships, several theories emerged in the late 20th century describing the different stages, the steps necessary to move from one stage to the next, and the deepening of interpersonal communication during this process (Mongeau & Henningsen, 2008). They differ mainly in their emphasis on specific components, for example on

communication (Knapp, 1978), self-disclosure (Altman & Taylor, 1973), or uncertainty reduction (Berger & Calabrese, 1975).

In **Figure S1**, four well-known theories of relationship development are presented and compared in terms of the relationship (sub)stages they contain: The *Conception of the Long-term Relationship* (Levinger, 1980), the *Staircase Model* (Knapp, 1978), the *Social Penetration Theory* (Altman & Taylor, 1973), and the *Uncertainty Reduction Theory* (Berger & Calabrese, 1975). In addition to these four theories, we also include the concept of maintenance behaviour (summarised by Ogolsky et al., 2017), which focuses on how partners act to maintain their relationship. As the different theories fit very well into Levinger's (1980) broader three stages, these are used as the basis for the classification, resulting in the stages of mutual attraction, building a relationship (or 'growing, satisfying continuation', as Levinger calls it), and maintaining a relationship after it has been declared.[1]

Figure S1

Stage/Process Models of Relationships

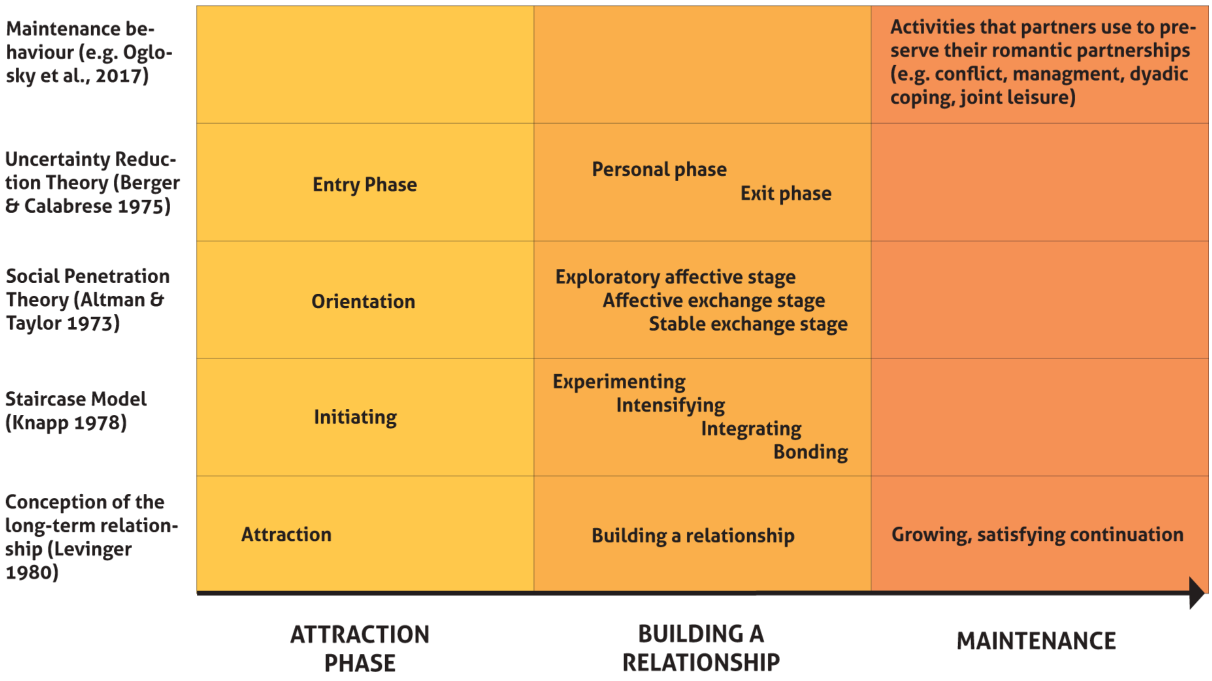

In the following, we will briefly describe the different relationship phases and discuss the factors that favour the occurrence of this stage and the progression to the next stage.

[1] In addition to these three phases, Levingers' model also includes the phases of deterioration and ending, which describe how relationships diverge. However, as the main focus of this paper is on how relationships are formed and maintained, these phases will not be described in detail.

Attraction

This phase involves the first encounter and the formation of first impressions by two individuals. It includes emotional and cognitive evaluations, as well as observable behaviours such as seeking closeness (e.g., Montoya & Horton, 2014). The emergence of mutual attraction plays a crucial role in determining the progression of a relationship to subsequent stages. Several internal and external factors influence this process, including both partners' personality traits, similarities in personality, values, and attitudes, closeness and familiarity, physical appearance, and reciprocal liking (Berscheid & Reis, 1998; Bode & Kushnick, 2021; Montoya & Horton, 2020).

In addition to the experience of attraction, this initial phase is characterised in the models presented by terms such as 'initiation', 'orientation', or 'entry', highlighting that the focus is on making initial contact and situating the social relationship for the first time. Communication during this stage is often superficial, guided by social norms or structured with limited personal disclosure (Altman & Taylor, 1973; Berger & Calabrese, 1975; Knapp, 1978).

Building a Relationship

The second stage of romantic love, extensively described in stage or process models, is characterised by increasing self-disclosure. During this process, communication becomes less superficial, which allows for a clearer understanding of each other and the disclosure of personal opinions and attitudes. To reduce uncertainty, various information-seeking strategies are employed, such as passive observation, active seeking of information from others, and interactive methods including direct questioning and mutual disclosure (Berger & Bradac, 1982). This leads to a state characterised by spontaneous communication and less influence of social desirability norms.

As well as gaining a deeper knowledge of each other, there is also a strengthening of mutual affective attachment and commitment in this phase. This involves the formation of a relational identity, the decisions about the future of the relationship, and may end with making the relationship public or taking significant steps such as marriage.

Maintaining a Relationship

While many of the models described end with the decision to enter into a relationship and communicate this to the outside world, such as the 'bonding' phase (Knapp, 1978) or the 'exit phase' (Berger & Calabrese, 1975), it is important to note that the relationship process does not end there, nor does the need for continued effort. For example, Berger and Calabrese (1975) point out that the 'exit phase' can occur several times, allowing for the renegotiation of whether the relationship should continue. From a biological perspective, the selection pressure for continuing a relationship may disappear after child rearing years are over, so exiting a relationship may make sense at this point (Fletcher et al., 2015). The relationship turbulence model (Solomon & Knobloch, 2004) suggests that turbulence, characterised by uncertainty about the relationship, can occur between the stages of casual dating and serious

relationship stages, as well as after entering into a committed relationship. Over time, therefore, relationship maintenance becomes crucial (Ogolsky et al., 2017). Maintenance strategies can generally be attributed to two primary motives: threat reduction, which involves managing conflict and addressing challenges, and relationship enhancement, which focuses on fostering connection and intimacy. Furthermore, these strategies can be implemented individually or through interactive efforts. Examples of individual strategies include positive illusions, idealisation, and gratitude, while interactive strategies include communication, conflict management, mutual support, humour, dyadic coping, and engaging in joint activities (Ogolsky et al., 2017).

Relationship Stages and Components of Love

There have been several efforts to link relationship stages to the triangular theory of love, often through cross-sectional designs that examine the manifestations of the components in relationships of varying durations. Acker and Davis (1992) found greater commitment in long-term relationships, declining passion in women, and relatively stable intimacy. Lemieux and Hale (2002), who included casual daters, showed that growth in commitment, intimacy and passion followed an inverted U pattern.

In addition, phase models based directly on component changes have been proposed. For example, García (1998) presents a three-phase model: 'being in love' with heightened erotic passion (around 6 months), 'passionate love' maintaining stable erotic passion while nurturing romantic passion, intimacy, and commitment (around 4 years), and 'companionate love' emphasising commitment and intimacy as passion wanes (typically around the fourth year). Similarly, Wojciszke (2002) outlines a six-phase framework, from intense 'falling in love' through 'romantic beginning' (passion and intimacy), 'complete love' (passion, intimacy, commitment), and 'companionate love' (intimacy and commitment) as the relationship matures. In addition, 'empty love' (commitment only) and 'dissolution' (absence of all components) phases suggest challenges and relationship decline. These phases align well with our model, where 'falling in love' corresponds to the first stage, 'romantic beginning' and 'complete love' to the second, and 'companionate love' to the third.

Overall, the empirical findings suggest that passion and intimacy become more important in the early stages, while passion becomes relatively less important over time and commitment becomes more important.

References

Acker, M., & Davis, M. H. (1992). Intimacy, passion and commitment in adult romantic relationships: A test of the Triangular Theory of Love. *Journal of Social and Personal Relationships*, *9*(1), 21–50. https://doi.org/10.1177/0265407592091002

Ainsworth, M. S. (1989). Attachments beyond infancy. *American Psychologist*, *44*, 709–716. https://doi.org/10.1037/0003-066X.44.4.709

Altman, I., & Taylor, D. A. (1973). *Social penetration: The development of interpersonal relationships* (pp. viii, 212). Holt, Rinehart & Winston.

Aristotle. (350 C.E., 1925). *Nicomachean ethics* (W. D. Ross, Trans.). Http://Classics.Mit.Edu/Aristotle/Nicomachaen.Html. http://classics.mit.edu/Aristotle/nicomachaen.html

Aron, A., & Tomlinson, J. M. (2018). Love as expansion of the self. In R. J. Sternberg & K. Sternberg (Eds.), *The New Psychology of Love* (2nd ed., pp. 1–24). Cambridge University Press. https://doi.org/10.1017/9781108658225.002

Baumeister, R. F., & Bratslavsky, E. (1999). Passion, intimacy, and time: Passionate love as a function of change in Intimacy. *Personality and Social Psychology Review*, *3*(1), 49–67. https://doi.org/10.1207/s15327957pspr0301_3

Berger, C. R., & Bradac, J. J. (1982). *Language and social knowledge: Uncertainty in interpersonal relations* (Vol. 2). Hodder Education.

Berger, C. R., & Calabrese, R. J. (1975). Some explorations in initial interaction and beyond: Toward. *Human Communication Research*, *1*(2), 99–112. https://doi.org/10.1111/j.1468-2958.1975.tb00258.x

Berscheid, E., & Reis, H. T. (1998). Attraction and close relationships. In D. T. Gilbert, S. T. Fiske, & G. Lindzey (Eds.), *The handbook of social psychology, Vols. 1-2, 4th ed* (pp. 193–281). McGraw-Hill.

Bode, A., & Kushnick, G. (2021). Proximate and Ultimate perspectives on romantic love. *Frontiers in Psychology*, *12*. https://www.frontiersin.org/articles/10.3389/fpsyg.2021.573123

Bowlby, J. (1999). *Attachment and loss* (2nd ed). Basic Books.

Campbell, L., & Ellis, B. (2005). Commitment, love, and mate retention. In *The Handbook of Evolutionary Psychology* (pp. 419–442). John Wiley & Sons, Inc.

Carswell, K. L., & Impett, E. A. (2021). What fuels passion? An integrative review of competing theories of romantic passion. *Social and Personality Psychology Compass*, *15*(8), e12629. https://doi.org/10.1111/spc3.12629

Fletcher, G., Simpson, J., Campbell, L., & Overall, N. (2015). Pair-bonding, romantic love, and evolution: The curious case of homo sapiens. *Perspectives on Psychological Science*, *10*, 20–36. https://doi.org/10.1177/1745691614561683

García, C. Y. (1998). Temporal course of the basic components of love throughout relationships. *Psychology in Spain*, *2*(1).

Gartner, C. A. (2017). Aristotle on love and friendship. In C. Bobonich (Ed.), *The Cambridge Companion to Ancient Ethics* (pp. 143–162). Cambridge University Press. https://doi.org/10.1017/9781107284258.009

Goode, W. J. (1959). The theoretical importance of love. *American Sociological Review*, *24*(1), 38–47. https://doi.org/10.2307/2089581

Goodman, C. (1999). Intimacy and autonomy in long term marriage. *Journal of Gerontological Social Work*, *32*(1), 83–97. https://doi.org/10.1300/J083v32n01_06

Hale, C. J., Hannum, J. W., & Espelage, D. L. (2005). Social support and physical health: The importance of belonging. *Journal of American College Health*, *53*(6), 276–284. https://doi.org/10.3200/JACH.53.6.276-284

Harasymchuk, C., Muise, A., Bacev-Giles, C., Gere, J., & Impett, E. (2020). Broadening your

horizon one day at a time: Relationship goals and exciting activities as daily antecedents of relational self-expansion. *Journal of Social and Personal Relationships*, *37*, 026540752091120. https://doi.org/10.1177/0265407520911202

Hatfield, E., Bensman, L., & Rapson, R. L. (2012). A brief history of social scientists' attempts to measure passionate love. *Journal of Social and Personal Relationships*, *29*(2), 143–164. https://doi.org/10.1177/0265407511431055

Hatfield, E., & Rapson, R. L. (1993). *Love, sex, and intimacy: Their psychology, biology, and history* (pp. xxii, 520). HarperCollins College Publishers.

Hatfield, E., & Sprecher, S. (1986). Measuring passionate love in intimate relationships. *Journal of Adolescence*, *9*(4), 383–410. https://doi.org/10.1016/s0140-1971(86)80043-4

Knapp, M. L. (1978). *Social intercourse: From greeting to goodbye*. Allyn and Bacon.

Kochar, R. K., & Sharma, D. (2015). Role of love in relationship satisfaction. *International Journal of Indian Psychology*, *3*(1). https://doi.org/10.25215/0301.102

Lee, J. A. (1973). *Colours of love: An exploration of the ways of loving* (First Edition). New Press.

Lemieux, R., & Hale, J. L. (2002). Cross-sectional analysis of intimacy, passion, and commitment: Testing the assumptions of the triangular theory of love. *Psychological Reports*, *90*(2), 1009–1014.

Levinger, G. (1980). Toward the analysis of close relationships. *Journal of Experimental Social Psychology*, *16*, 510–544. https://doi.org/10.1016/0022-1031(80)90056-6

Lewis, C. S. (2012). *Four Loves*. Collins.

Mongeau, P. A., & Henningsen, M. L. M. (2008). Stage theories of relationship development. *Engaging Theories in Interpersonal Communication: Multiple Perspectives*, *363*, 375.

Monier-Williams, S. M., Leuman, E., Cappeller, C., Monier-Williams, S. M., Leuman, E., &

Cappeller, C. (1963). *A Sanskrit-English dictionary: Etymologically and philologically arranged with special reference to cognate Indo-European languages*. Oxford University Press.

Montoya, R. M., & Horton, R. S. (2014). A two-dimensional model for the study of interpersonal attraction. *Personality and Social Psychology Review*, *18*(1), 59–86. https://doi.org/10.1177/1088868313501887

Montoya, R. M., & Horton, R. S. (2020). Understanding the attraction process. *Social and Personality Psychology Compass*, *14*(4), e12526. https://doi.org/10.1111/spc3.12526

Moss, B. F., & Schwebel, A. I. (1993). Defining intimacy in romantic relationships. *Family Relations*, *42*(1), 31. https://doi.org/10.2307/584918

Ogolsky, B. G., Monk, J. K., Rice, T. M., Theisen, J. C., & Maniotes, C. R. (2017). Relationship maintenance: A review of research on romantic relationships. *Journal of Family Theory & Review*, *9*(3), 275–306. https://doi.org/10.1111/jftr.12205

Patrick, S., Sells, J. N., Giordano, F. G., & Tollerud, T. R. (2007). Intimacy, differentiation, and personality variables as predictors of marital satisfaction. *The Family Journal*, *15*(4), 359–367. https://doi.org/10.1177/1066480707303754

Rubin, H., & Campbell, L. (2012). Day-to-day changes in intimacy predict heightened relationship passion, sexual occurrence, and sexual satisfaction: A dyadic diary analysis. *Social Psychological and Personality Science*, *3*(2), 224–231.

Rusbult, C. E., & Buunk, B. P. (1993). Commitment processes in close relationships: An interdependence analysis. *Journal of Social and Personal Relationships*, *10*(2), 175–204. https://doi.org/10.1177/026540759301000202

Sneed, J. R., Whitbourne, S. K., Schwartz, S. J., & Huang, S. (2012). The relationship between identity, intimacy, and midlife well-being: Findings from the Rochester Adult Longitudinal Study. *Psychology and Aging*, *27*(2), 318–323.

https://doi.org/10.1037/a0026378

Solomon, D. H., & Knobloch, L. K. (2004). A model of relational turbulence: The role of intimacy, relational uncertainty, and interference from partners in appraisals of irritations. *Journal of Social and Personal Relationships*, *21*(6), 795–816. https://doi.org/10.1177/0265407504047838

Sorokowski, P., Sorokowska, A., Karwowski, M., Groyecka, A., Aavik, T., Akello, G., Alm, C., Amjad, N., Anjum, A., Asao, K., Atama, C. S., Atamtürk Duyar, D., Ayebare, R., Batres, C., Bendixen, M., Bensafia, A., Bizumic, B., Boussena, M., Buss, D. M., … Sternberg, R. J. (2021). Universality of the triangular theory of love: Adaptation and psychometric properties of the triangular love scale in 25 countries. *The Journal of Sex Research*, *58*(1), 106–115. https://doi.org/10.1080/00224499.2020.1787318

Sternberg, R. J. (1986). A triangular theory of love. *Psychological Review*, *93*, 119–135. https://doi.org/10.1037/0033-295X.93.2.119

Wieselquist, J., Rusbult, C. E., Foster, C. A., & Agnew, C. R. (1999). Commitment, pro-relationship behavior, and trust in close relationships. *Journal of Personality and Social Psychology*, *77*(5), 942–966.

Wojciszke, B. (2002). From the first sight to the last drop: A six-stage model of the dynamics of love. *Polish Psychological Bulletin*, *33*, 15–25.
